# Crack Healing Performance of PVA-Coated Granules Made of Cement, CSA, and Na_2_CO_3_ in the Cement Matrix

**DOI:** 10.3390/ma9070555

**Published:** 2016-07-09

**Authors:** Yong-Soo Lee, Jae-Suk Ryou

**Affiliations:** Department of Civil & Environmental Engineering, Hanyang University, 222 Wangsimni-ro, Seongdong-gu, Seoul 133-791, Korea; imcivil@hanyang.ac.kr

**Keywords:** self-healing, cementitious materials, granulation, coating, PVA film

## Abstract

Various self-healing methods for concrete, such as the use of supplementary cementitious materials, adhesive agents, mineral admixtures, and bacteria, have been suggested to date, and each of these has merits and demerits. Among these, however, the use of cementitious materials may be appropriate due to their good healing efficiency, low cost, and compatibility with the cement matrix. In this study, granulation and coating methods were applied to a new cementitious composite material. The self-healing property of these materials was controlled by the polyvinyl alcohol (PVA) coating until cracks were created. Water dissolved the PVA coating after entering through the cracks, and reacted with the healing materials to generate healing products. The self-healing performance was evaluated at various elapsed times through the measurement of the crack widths, visual observation, and examination of the microscopic images. Simultaneously, a water permeability test was performed and the dynamic modulus of elasticity was measured to verify the recovery of the cracks. In addition, the healing products that had been formed in the cracks were analyzed via X-ray diffraction (XRD) and scanning electron microscopy (SEM).

## 1. Introduction

Concrete is a widely used construction material because of its numerous advantages, such as its strength, low cost, and ease of manufacture as well as the possibility of constructing structural members with numerous shapes and sizes with it. It also has some disadvantages, however, such as its low tensile strength and the appearance of cracks therein. Various types of cracks can arise in plain and reinforced concrete structures, and it is difficult to prevent their occurrence [1,2]. They give access to the harmful ions present in the environment, such as chloride, carbon dioxide, oxygen, sulfates, salts, and water, allowing them to enter the concrete and to attack the reinforcing bars and cement paste. These attacks cause corrosion of the steel as well as expansion and enlargement of the existing cracks on the concrete. To increase the service life of concrete structures, periodic maintenance is needed, but the maintenance costs are generally high, and repair works can interrupt the serviceability of a structure like a bridge, tunnel, or building for the public [3]. For underwater and underground structures, man-made repair is difficult or even impossible due to their inaccessibility [4]. The self-repair of the cracks by the concrete has been perceived as a novel solution for reducing the life cycle cost (LCC) and increasing the service life of concrete structures. Additionally, such a concrete has eco-friendly advantages, such as material savings, reduction of the pollution and energy consumption during the construction, and low CO_2_ emission [3,5].

The natural healing of the cracks in concrete is an inherent property as the micro cracks in concrete may be filled with products from the further hydration of the unhydrated cement and the crystals of the CaCO_3_ produced due to the reaction of the CO_2_ with the moisture present in the concrete environment [6], but natural healing is a slow process, and the recovery of the cracks is incomplete. Improving the self-healing efficiency of concrete has drawn attention worldwide for the last two decades. Many related experimental studies and explorations have been conducted, such as the use of supplementary cementitious materials, adhesive agents, mineral admixtures, and bacteria and the embedment of devices like microcapsules, brittle glass pipes, and shape memory materials in concrete to accelerate the filling of cracks. JCI Technical Committee TC-075B has classified the terminologies related to self-healing [7]. Various review papers have critically summarized the above approaches of self-healing [4,8,9,10,11].

The mineral admixtures that are used as self-healing materials can be divided into two groups: (1) expansive mineral admixtures (i.e., calcium sulfo aluminate (4CaO·3Al_2_O_3_·SO_3_), free lime, and anhydrite (CaSO_4_)); and (2) crystalline mineral admixtures (i.e., Na_2_CO_3_, and NaHCO_3_) [12,13]. These admixtures react with the Ca(OH)_2_ present in concrete to make crystal products. Expansive mineral admixtures react with the penetrating water through a crack and create products in the crack. These products have a volume larger than the admixture itself, which is why they are called “expansive admixtures”. Calcium sulfo aluminate (CSA) has been used as a self-healing material in previous studies [14,15,16] for filling cracks. These admixtures are low-cost and have low compatibility with cement paste, but some studies [15,17] have shown that when they are directly added to concrete during mixing, they start reacting with water and can cause unwanted expansion in the concrete. At a later stage, when the concrete cracks and such admixtures are needed for the self-healing of the cracks, their amount will be deficient to generate enough healing products to fill the cracks. A solution for preventing the formation of expansive products and the early undesirable use of these minerals is to first encapsulate them and then add these capsules to the concrete. Encapsulation can be beneficial as it is effective due to the response of the microcapsules dispersed in the concrete to the many damage locations at the same time. These microcapsules may release the healing agent only when needed or when ruptured by a crack. It is possible, however, for a crack to hit a microcapsule but to deviate along the interface of the microcapsule and cement paste instead of intersecting it [4]. In such a circumstance, the encapsulated material will not be available for self-healing. Additionally, it is difficult to prepare and cast the capsules in concrete. The compatibility and bonding between the capsules and the matrix is another concern [4,11,14]. Table 1 compares the characteristics of the microcapsule and expansive mineral agents in self-healing [4].

### Self-Healing through Granulation and Film Coating

Granulation is the process of making granules from powder by first moistening the powder and then passing the moist powder through a screen with a proper mesh size. This causes bonding to start between the particles of the wet granules, and for them to get interlocked. The granules are then air- or heat-dried in a vibrating pan to prevent their adhesion into a large mass. This technique is used in the pharmaceutical industry for the production of medicines [18,19,20].

The film-coating process, which places a thin, skin-tight coating of a polymer material over the solid preparations (e.g., the tablets and granules), was developed to produce coated solid preparations with essentially the same weight, shape, and size as the original solid preparations. Solid preparations are film-coated by applying or spraying the coating solution on them in ordinary coating pans. The volatility of the solvent enables the film to adhere quickly to the surface of the solid preparation. To allow a gradual build-up of the coating, the solution is added in portions, with warm air blown in to hasten the drying [21].

In the previous study [22], a calcium sulfo aluminate (CSA)-based admixture was first granulated and then coated with a film of polyvinyl alcohol (PVA) with two aims: (1) to control the response time of the healing material; and (2) to combine the advantages of granulation and film coating. The CSA-based mineral admixture contained hauyne (3CaO·3Al_2_O_3_·CaSO_4_), free lime, and anhydrite (CaSO_4_). The granules were used as a fraction replacement of cement in the mortar. Cracks were induced in the mortar prism, and their self-healing efficiency was studied. Crack closing can be achieved by healing products that form ettringite and calcium carbonate, but due to the early formation of less stable ettringite, which has a low density, the internal cracks could not be filled completely. Therefore, the healing performance may be limited.

In this study, the self-healing performance of a new cementitious composite admixture was studied. This cementitious composite contains three materials: ordinary cement, a CSA-based expansive admixture, and sodium carbonate (Na_2_CO_3_). They were granulated and coated with PVA film. Their self-healing performance was evaluated by measuring the crack widths and through microscopic investigation at different time intervals. Simultaneously, a water permeability test was performed, and the dynamic modulus of elasticity was measured. In addition, scanning electron microscopy (SEM) and X-ray diffraction (XRD) were carried out to analyze the healing products that were formed in the cracks.

## 2. Experiment Procedure

### 2.1. Granulation/Coating Materials

**Figure 1:**
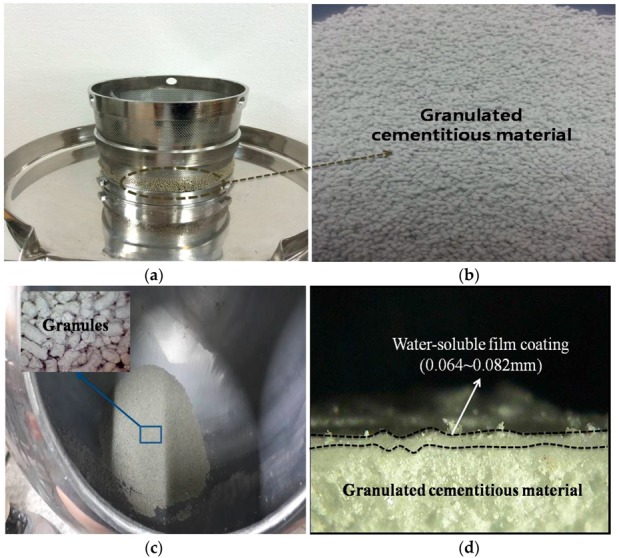
(**a**) Granulating machine; (**b**) a closer view of the granules obtained from the granulating machine; (**c**) granules tumbling over one another in the rotating pan where they were dried with hot air before they were sprayed with PVA solution; and (**d**) the PVA coating of the granules.

The granulation materials and mixture proportions in this study are shown in Table 2. They were mixed together, moistened with pure water, kneaded, and then put in a granulating machine, where they were forced to pass through a screen for making granules. The obtained granules were dried with hot air in a tray and then transferred to an acorn-shaped stainless steel pan with a 1 ft diameter. This pan operates at a 40° angle and is open in front for visual and manual access by the operator. The pan was rotated at a moderate speed, and the granules started tumbling over one another. The PVA- and purified-water-blended solution was sprayed onto the granules inside the pan, and then hot air was simultaneously blown on them. The surfaces of the granules were coated with the PVA while the purified water evaporated [23]. The final granules were cylindrical in shape, with a 0.3–0.5 mm diameter and length and a 0.1963 mm^3^ volume. The PVA film coating was 0.064–0.028 mm thick. Figure 1 shows the granulation process of the cementitious material and the granules coated with the water-soluble film in a pan.

### 2.2. Preparation of Specimens for Evaluating the Healing Efficiency

Mortar mixtures were made to evaluate the healing efficiency, based on a 0.4 w/c ratio and a 1:3 cement-to-sand ratio, according to KS L ISO 679 [24]. The total cement replacement with PVA-coated granules was limited to 10% to avoid unexpected expansion [22]. The healing efficiency was investigated at six levels, as shown in Table 2 (Granule-EA, Granule-Cm#1, Granule-Cm#2, Granule-Cm#3, Granule-Cm#4, and Granule-Cm#5), and the values that were obtained were compared with that of the plain specimen. EA shows the granulated CSA-based expansive agent with a PVA film coating, and Cm is the granulated cementitious material (cement + CSA-based expansive agent + sodium carbonate). The number after Cm indicates the ratio of the cement to the expansive agent. The replacement ratio of sodium carbonate was fixed at 1%. Insoluble calcium carbonate (CaCO_3_) was formed by the reaction of sodium carbonate (Na_2_CO_3_) to calcium hydroxide (Ca(OH)_2_), which was formed by the C_3_S and β-C_2_S hydration, and from the CSA-expansive agent [25]. Calcium carbonate is known as the main product for filling crack faces. Table 2 shows the details of the experimental mixture. 

In this study, all the mortar mixtures were placed in 40 mm × 40 mm × 160 mm molds, in different layers. First, a 10 mm mortar layer was brought into molds and was compacted via vibration. Two Ф2 mm reinforcement bars were placed on this layer to avoid complete fracture while introducing a crack through a three-point bending machine. Afterwards, the molds were further filled with mortar. To evaluate the healing efficiency of the mortar specimens, however, they were cured at 20 ± 2 °C in a 95% ± 5% humidity chamber until the testing time.

**Figure 2:**
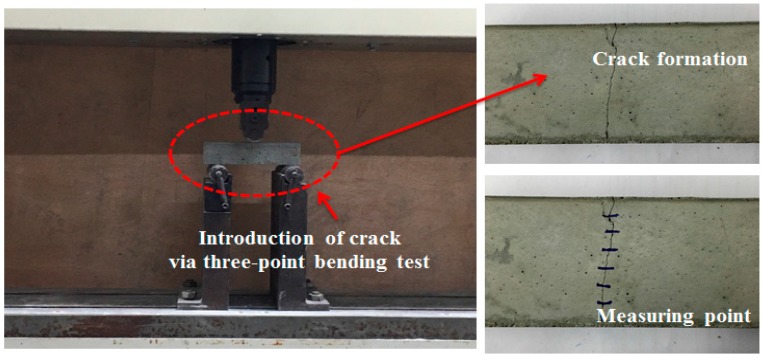
View of the three-point bending test for crack introduction, and view of the crack from the lower end of the mortar prism.

Cracks may range from very small internal microcracks that occur during the application of modest amounts of stress to large cracks caused by undesirable interactions with the environment, poor construction practices, or errors in structural design and detailing. These cracks can be classified into microcracks (width = below 0.1 mm), mesocracks (width = 0.1–0.7 mm), and macrocracks (width = over 0.7 mm). For the durability of concrete, it is important to keep the crack width within the allowable range by uniformly distributing microcracks instead of macrocracks. The crack width needed for durability is limited to below 0.3 mm. Also, the crack width that cannot be exposed to the risk of water leakage is usually 0.06 mm. In particular, the crack width for waterproofing should be below 0.2 mm because water leakage undoubtedly occurs at over 0.2 mm [26]. Thus, after 28 days of curing, the samples were removed, and cracks ranging from 0.01 to 0.35 mm were created using a three-point bending machine, as shown in Figure 2. The cracks were measured at a minimum of six different points along the crack length, and then the average of the obtained values was taken as the crack width. The specimens were loaded up to 80% failure in the three-point bending machine. The load limit was selected through various experiment trials to produce cracks within the range mentioned.

### 2.3. Evaluation of the Healing Efficiency

To evaluate the healing ability of the self-healing material or the improvement of its healing performance, it is necessary to compare the properties of the self-healing material before and after the carrying out of its healing functions. Many techniques and parameters have been used to compare such properties quantitatively and qualitatively. In particular, microscopic investigation is a common qualitative evaluation method [7]. In this study, all the cracked specimens were immersed in water, and then the crack widths and images were obtained via microscopic investigation depending on the elapsed time after drying the specimens. It is also often desirable to perform a non-destructive test of the stiffness of a concrete specimen so that the progressive changes in the material due to sustained chemical attack, the repeated freezing–thawing cycles, the aging, or other factors can be measured. In particular, for the evaluation of the degree of damage that occurred inside the concrete (the microcrack inside the concrete), dynamic modulus of elasticity measurements are very convenient [27].

While the recovery of the crack on the surface of the specimen can be monitored with a microscope, the crack inside the specimen cannot. Therefore, the dynamic modulus of elasticity was measured along with the conduct of microscopic investigation, according to ASTM C215 [28]. Subsequently, the achievement of internal crack closing was evaluated based on the relative dynamic modulus of elasticity using the following equation:

(1) $$P_{c}=\frac{\left[{n_{c}}^{2}\right]}{\left[{n_{0}}^{2}\right]}\times 100\;,$$

where *P_c_* denotes the relative dynamic modulus of elasticity depending on the elapsed time (%), *n*_0_ shows the 1st horizontal vibration before the crack introduction, and *n_c_* shows the horizontal vibration frequency after the crack introduction depending on the elapsed time.

**Figure 3:**
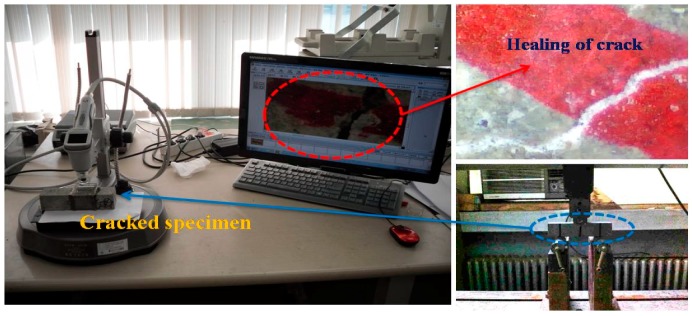
Investigation of crack width with a microscope.

The relative dynamic modulus of elasticity was determined based on the percentage of the dynamic modulus of elasticity before and after the crack introduction depending on the elapsed time. The equipment that was used for the measurement of the relative dynamic modulus of elasticity was MIN-011-0-3S from MARVI & Co., Ltd. (Seoul, Korea). In addition, the occurrence of internal crack closing was determined through the use of a microscope after cutting the surface of the specimen 10 mm deep using a cutter. Figure 3 shows the investigation of the crack width with a microscope.

**Figure 4:**
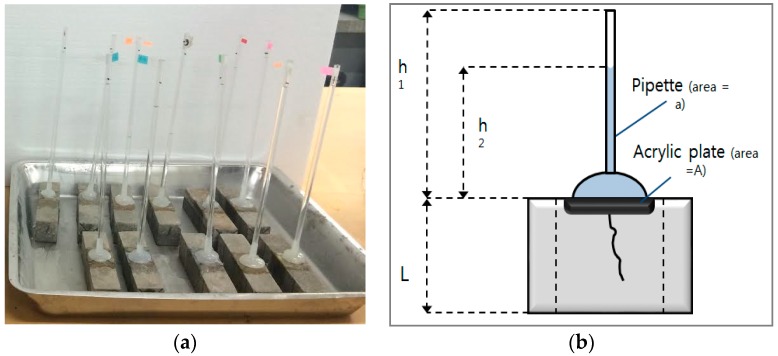
Schematic view of the water permeability test. (**a**) Water permeability test being conducted on cracked mortar prism beams; (**b**) longitudinal-cross-sectional view of these specimen under test.

The improvement by a healing function is more pronounced in a transport substance such as ion or water [2,29]. To evaluate the prevention of aggressive agents such as the migration of water due to crack propagation, a water passing test was conducted, apart from microscopic investigation. The instrument design that was adopted was similar to that which was used in RILEM Test Method II.4 [30], as shown in Figure 4. The water permeability coefficient was calculated using Equation (2) derived by Cernica [31].

(2) $$k=\frac{aL}{At}\ln[\frac{h_{1}}{h_{2}}],$$

where *k* = water permeability coefficient (cm/s); *a* = cross-sectional area of the pipette (cm^2^); *L* = specimen thickness (cm); *A* = cross-sectional area of the specimen (cm^2^); *T* = time (s); *h*_1_ = initial water head (cm); and *h*_2_ = final water head (cm).

In this study, a scanning electron microscope with a 0.2–30 kV accelerating voltage, a 10^−12^–10^−5^ A probe current, a 3.5 mm SEI resolution (WD = 8 mm; Acc = 35 kV), 10–3,000,000× magnifications, and SEI, BEI, and X-ray image modes was used to evaluate the healing products. Samples conforming to the report were obtained for the SEM analysis [32]. For the XRD analysis, however, an apparatus with CuK (Ni, filter), 35 kV, 20 mA, 2°/min scan speed, full scale, 140 cps, and 2θ = 5°–40° specifications was used, and samples were obtained by scraping off part of the healing product after complete crack closing. The specimens were oven-dried before sample extraction for XRD analysis. After the extraction of the healing products that were formed within the cracks, the samples were kept vacuum-tight until analysis was carried out to avoid further reaction. Microscopic images of the crack widths were obtained using a video microscope system (EGVM 452M) from EG TECH Korea (Seoul, Korea), with a 4300X maximum modification range.

## 3. Results and Discussion

### 3.1. Microscopic Investigation of the Changes in the Crack Width

**Figure 5:**
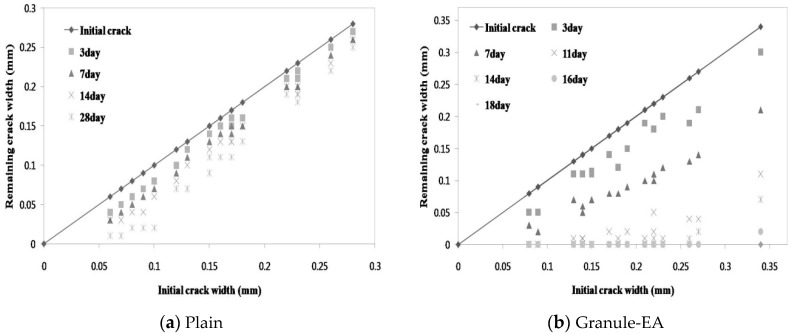

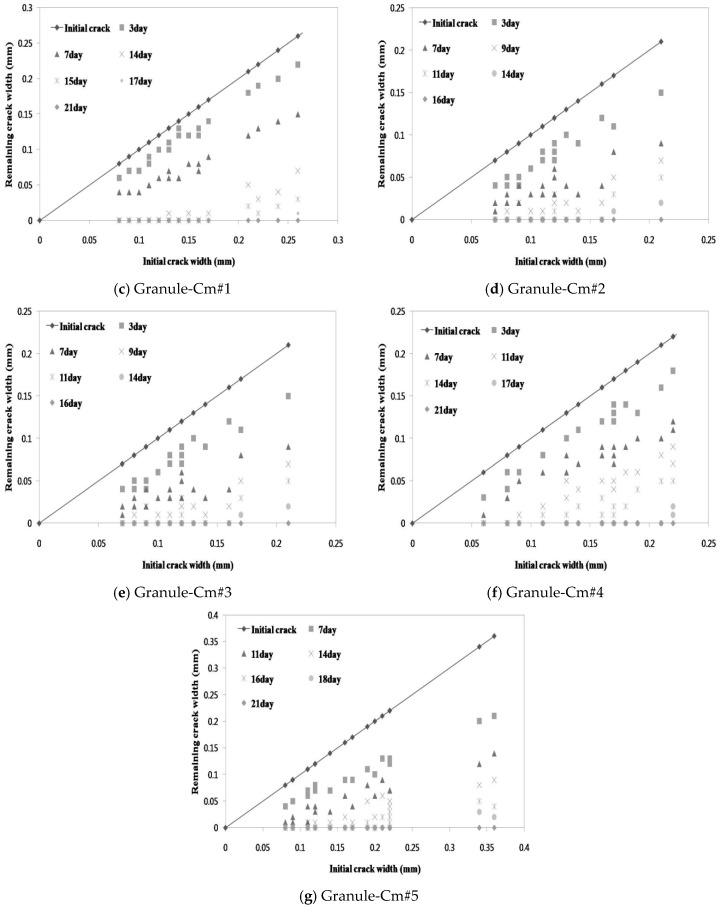
Changes in the crack width at each observation day: (**a**) Plain; (**b**) Granule-EA (CSA-based expansive agent); (**c**) Granule-Cm#1; (**d**) Granule-Cm#2; (**e**) Granule-Cm#3; (**f**) Granule-Cm#4; and (**g**) Granule-Cm#5.

Crack width images were obtained via microscopic observation at each specified time interval. Figure 5 shows the changes in the crack width of each mixture (Plain, Granule-EA, Granule-Cm#1, Granule-Cm#2, Granule-Cm#3, Granule-Cm#4, and Granule-Cm#5) with respect to the elapsed time. In Figure 5, Plain shows that crack closing was not completely achieved in all the crack widths up to the 28 days elapsed time. Although the <0.1 mm crack width was not completely closed, as reported in the previous research [6], it showed a similar crack closing. For the granulated CSA-based expansive agent with a PVA film coating (Granule-EA), the <0.1 mm, 0.1–0.2 mm, and >0.2 mm crack widths indicated complete crack closing within 11, 14, and 16 days, respectively. For the granulated cementitious material with a PVA film coating (Granule-Cm), the <0.1 mm crack widths indicated complete crack closing within 14, 11, 11, 14, and 14 days, respectively, in the following order: #1, 2, 3, 4, and 5. The 0.1–0.2 mm crack widths indicated complete crack closing within 15, 14, 14, 17, and 16 days, respectively. Finally, the >0.2 mm crack widths indicated complete crack closing within 21, 16, 21, and 21 days, respectively, in this order: #1, 2, 3, 4, and 5. It was noticed that the 0.2–0.3 mm crack widths of Granule-Cm#5 indicated complete crack closing within 18 days.

Therefore, it was verified via microscopic investigation that all the mixtures, except Plain, showed complete crack closing within 21 days. In addition, the time of the crack closing of Granule-EA (the CSA-based expansive agent) was slower than that of Granule-Cm (the cement + CSA-based expansive agent + sodium carbonate). This indicates that the formation rate of the healing products formed on the crack faces by the hydration of the healing material (the CSA-based expansive agent and the cementitious material) was different. Therefore, it was verified that the interior-healing material might have reacted with the water molecules as the water that migrated through the cracks dissolved the water-soluble PVA film and crack closing was achieved.

**Figure 6:**
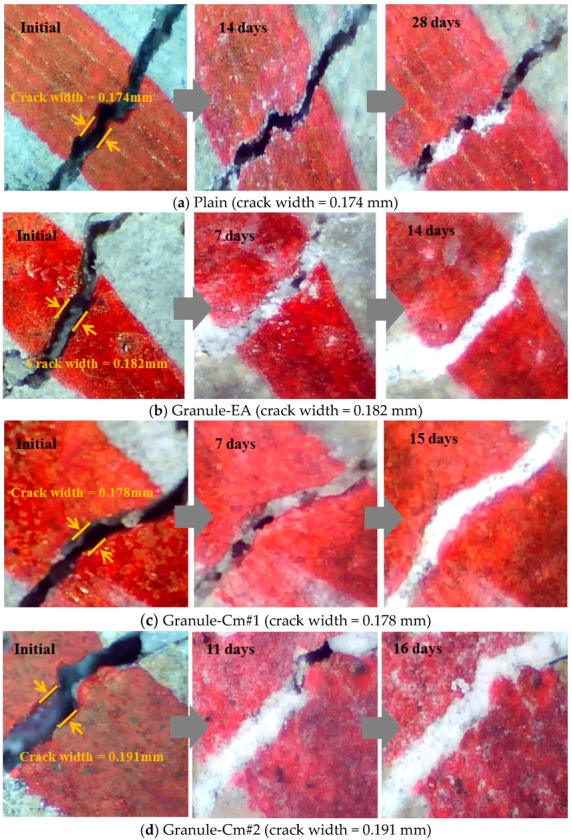

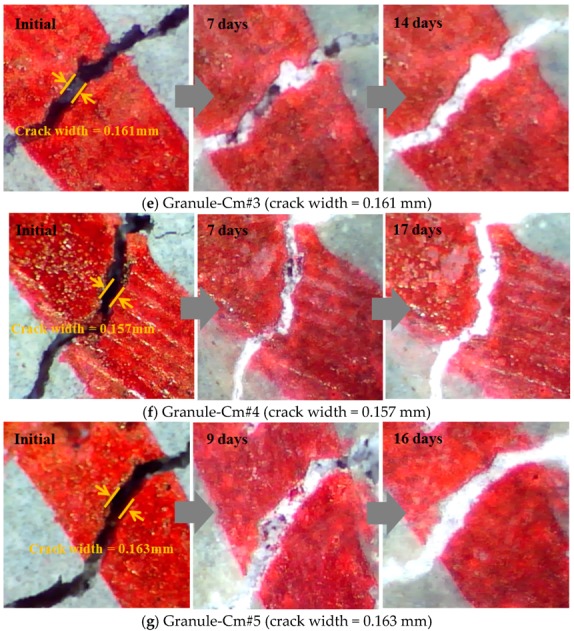
Crack closing process due to self-healing: (**a**) Plain; (**b**) Granule-EA (CSA-based expansive agent); (**c**) Granule-Cm#1; (**d**) Granule-Cm#2; (**e**) Granule-Cm#3; (**f**) Granule-Cm#4; and (**g**) Granule-Cm#5.

Figure 6 shows the process of crack closing via self-healing with regard to the typical specimens Plain and the granules with the PVA film coating (Granule-EA and Granule-Cm), which involved 0.1–0.2 mm crack widths within the crack width range. Plain in Figure 6 shows the occurrence of crystals on both faces of the crack after 14-day immersion, and crack closing was observed in some parts of the crack after 28-day immersion. As the specimens were dried before the microscopic investigation, these crystals were assumed to have been CaCO_3_, which could be formed by the action of the leached Ca(OH)_2_ out of the cracks and the CO_2_ in the air [6]. The specimen that incorporated Granule-EA was observed to have had crystals, except for some parts of the crack, after seven-day immersion. It also showed complete crack closing after 14-day immersion. Therefore, it can be supposed that crack closing was achieved because the CSA-based expansive agent had reacted due to the removal of the PVA film coating by the water that migrated through the crack faces. 

For Granule-Cm, although the specimens that incorporated it (Granule-Cm#1, Granule-Cm#2, Granule-Cm#3, Granule-Cm#4, and Granule-Cm#5) were similar to those in the crack closing process of Granule-EA, the time of their complete crack closing differed. As shown in Figure 6c–g, after seven-day immersion, the extent of the crack closing of the specimens that incorporated Granule-Cm was smaller than that of Granule-EA. Moreover, complete crack closing was observed after 14- to 17-day immersion. This can be supposed as indicative that the cementitious material, used as the healing material, had formed various hydration products on the crack faces. Therefore, the microscopic images verified that the healing material had reacted after the removal of the PVA film coating by the migrated water via the crack faces.

### 3.2. Evaluation of the Internal Crack Closing via the Dynamic Modulus of Elasticity

While the recovery of the crack on the surface of the specimen could be monitored with a microscope, the crack inside the specimen could not. Therefore, the dynamic modulus of elasticity was measured in conjunction with the microscopic investigation. Subsequently, the internal crack closing was evaluated based on the relative dynamic modulus of elasticity, which was determined based on the percentage of the dynamic modulus of elasticity before and after the crack introduction depending on the elapsed time.

**Figure 7:**
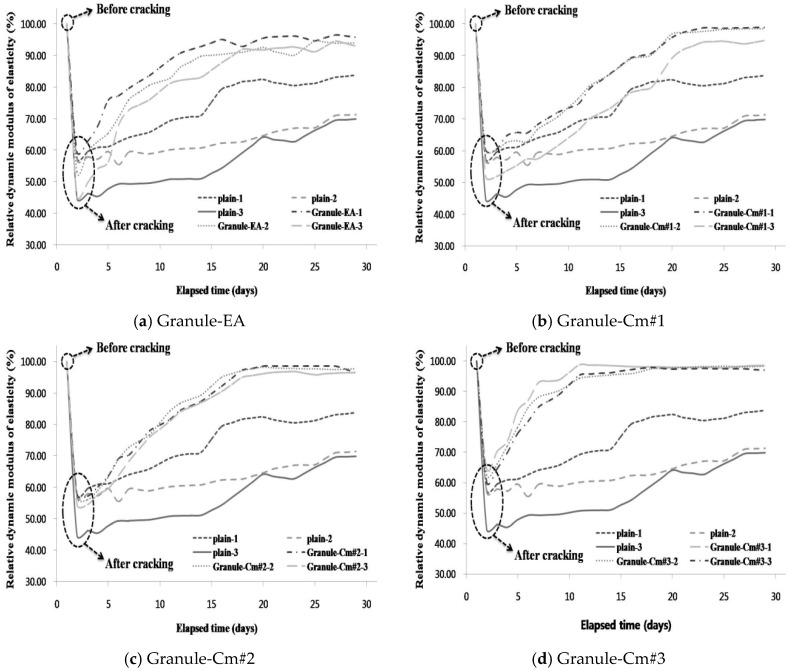

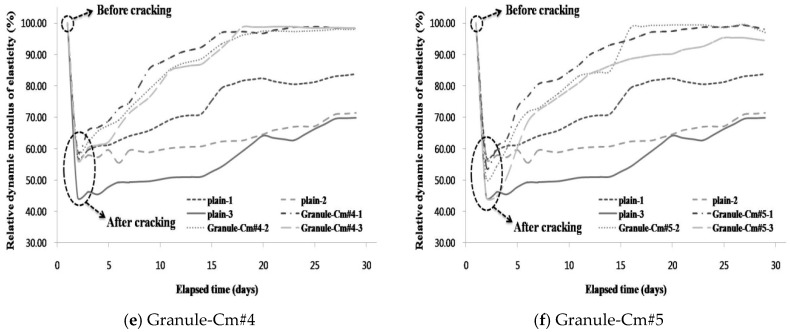
Comparison of the relative dynamic modulus of elasticity values of the mixtures: (1: <0.1 mm; 2: 0.1–0.2 mm; 3: >0.2 mm): (**a**) Granule-EA (CSA-based expansive agent); (**b**) Granule-Cm#1; (**c**) Granule-Cm#2; (**d**) Granule-Cm#3; (**e**) Granule-Cm#4; and (**f**) Granule-Cm#5.

Figure 7 shows the relative dynamic modulus of elasticity in each mixture compared with that in Plain. Plain-1 represents the crack widths within the range less than 0.1 mm. In the same way, Plain-2 and Plain-3 show the crack widths with the 0.1–0.2 mm and greater than 0.2 mm ranges, respectively. As can be seen in Figure 7, the mean relative dynamic modulus of elasticity in all the specimens showed a sharp decrease when the cracks appeared. The mean relative dynamic modulus of elasticity of Plain-1 reached about 80% of the state before the introduction of cracks, with a gradual increase depending on the elapsed time, and those of Plain-2 and Plain-3 reached about 60%. These indicate that Plain did not achieve complete internal crack closing. In Figure 6a, Granule-EA-1, Granule-EA-2, and Granule-EA-3 show over 90% values for the mean relative dynamic modulus of elasticity, with a rapid increase from five days depending on the crack width range. Although a 100% dynamic modulus of elasticity was not reached, it can be supposed that complete internal crack closing was almost achieved. In Figure 6b–f, the mean relative dynamic modulus of elasticity of Granule-Cm shows a pattern different from that of Granule-EA in the early stage. The mean relative dynamic modulus of elasticity of Granule-EA increased rapidly early then stabilized whereas that of Granule-Cm increased gradually and then stabilized. This is because the formation rates of the healing products that were formed by the healing material differed. In particular, the recovery of the relative dynamic modulus of elasticity of Granule-Cm#2 and Granule-Cm#3 were the most excellent. It should also be noted that Granule-Cm#2 and Granule-Cm#3 are superior to Granule-EA.

**Figure 8:**
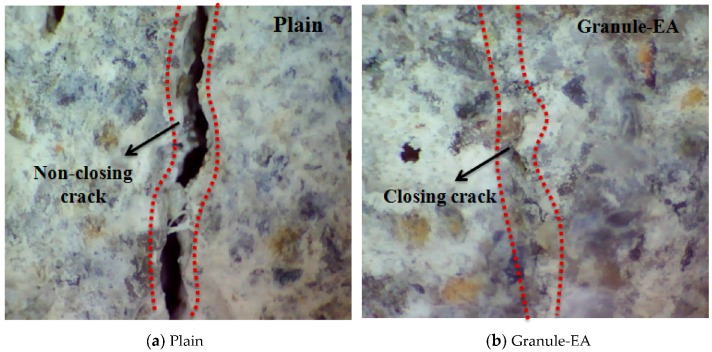

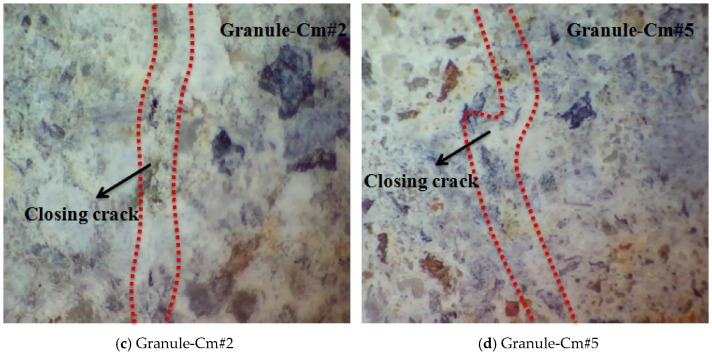
Microscopic image of the specimen inside after the completion of the test: (**a**) Plain; (**b**) Granule-EA (CSA-based expansive agent); (**c**) Granule-Cm#2; and (**d**) Granule-Cm#5.

After the completion of the measurement of the dynamic modulus of elasticity, the presence of internal crack closing was observed with a microscope after the surface of the specimen was cut 10 mm deep using a cutter. Figure 8 shows a microscopic image of the specimen inside. As Granule-Cm showed a similar internal crack closing, Granule-Cm#2 and Granule-Cm#5 were observed with a microscope, respectively. In Figure 8, Plain is shown to have hardly achieved internal crack closing. On the other hand, Granule-EA and -Cm are shown to have achieved internal crack closing because marks of the cracks could no longer be observed. This can be taken to mean that internal crack closing was achieved without difficulty because the crack width of the inside of the specimen was smaller than the width (0.1–0.2 mm) of the crack that appeared on the surface. Therefore, the self-healing efficiency of the concrete was verified on the internal crack of the specimen.

### 3.3. Evaluation of the Prevention of Water Migration by the Water Permeability Coefficient

**Figure 9:**
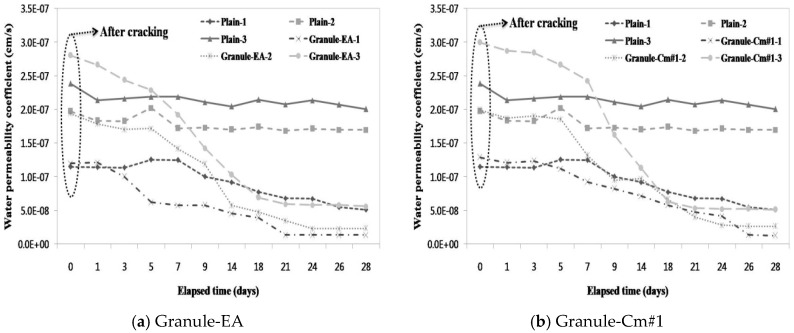

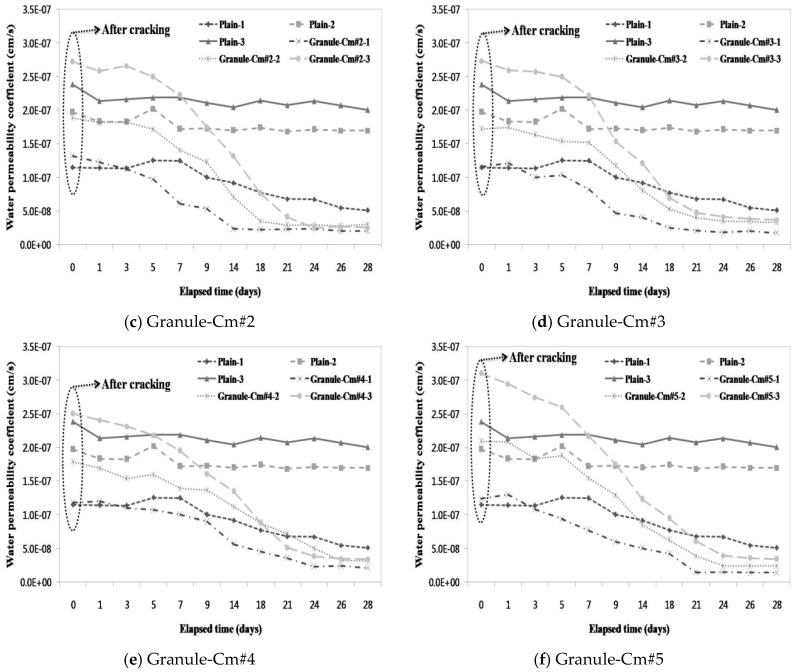
Water permeability coefficient on each crack width range in each mixture (1: <0.1 mm; 2: 0.1–0.2 mm; 3: >0.2 mm): (**a**) Granule-EA (CSA-based expansive agent); (**b**) Granule-Cm#1; (**c**) Granule-Cm#2; (**d**) Granule-Cm#3; (**e**) Granule-Cm#4; and (**f**) Granule-Cm#5.

A water passing test was conducted parallel to the microscopic investigation to evaluate the prevention of aggressive agents such as water migration due to crack propagation. Subsequently, the water permeability coefficient in each mixture was calculated. Figure 9 compares the water permeability coefficient on each crack width range in each mixture with that in the other mixtures. Figure 9 shows that a similar water permeability coefficient was obtained for each crack width range (<0.1 mm, 0.1–0.2 mm, and >0.2 mm). In particular, unlike the aforementioned crack closing results, the water permeability coefficients of Plain-2 and Plain-3 did not significantly differ in state after the introduction of cracks, and that of Plain-1 only slightly decreased.

As shown in Figure 9a, the water permeability coefficient of Granule-EA significantly decreased with the increase in the immersion period. In particular, although the water permeability coefficient differed at the initial crack, Granule-EA-1 and Granule-EA-2 showed similar values after 24 days. In addition, the water permeability coefficient of Granule-EA-3 was similar to that of Plain-1, and, unlike Granule-EA, the water permeability coefficient of Granule-Cm gradually decreased up to seven days, and then rapidly decreased. This shows the differences in the formation rates of the healing products, which were formed on the crack faces by the hydration of the healing material.

As shown in Figure 9b–f, Granule-Cm, except for Granule-Cm#1–3, had a low water permeability coefficient compared to Plain-1 at 28 days. Although the water permeability coefficient of most of the mixtures decreased depending on the crack width range, that of Granule-Cm#2 hardly changed regardless of the crack width range. It should be noted that Granule-Cm#2’s resistance to permeability was the most excellent, as shown in the results for the relative dynamic modulus of elasticity. Therefore, the efficacy of the crack closing, which prevents water migration through the self-healing of the granule with a PVA film coating, was verified.

### 3.4. Analysis of the Healing Products Formed on the Crack Faces

**Figure 10:**
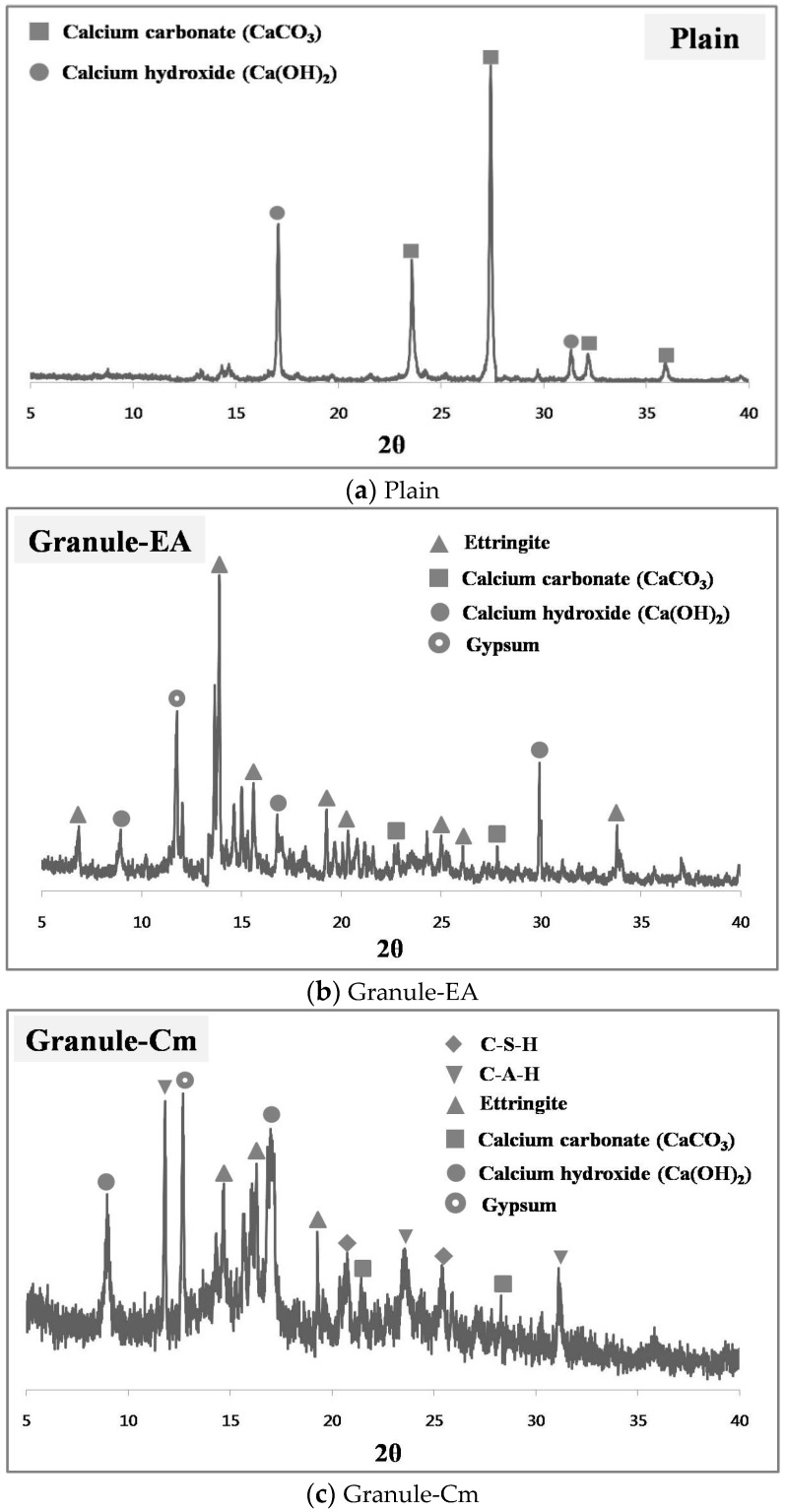
XRD pattern of the healing products in the crack face: (**a**) Plain; (**b**) Granule-EA; and (**c**) Granule-Cm.

SEM and XRD tests were conducted to analyze the healing products that were formed within the cracks. Figure 10 shows the XRD patterns of the healing products of Plain (with no healing material incorporated), the specimens that incorporated the granulated CSA-based expansive agent with the PVA film coating (Granule-EA), and the granulated cementitious material (the cement + CSA-based expansive agent + sodium carbonate) with the PVA film coating (Granule-Cm#2). In Figure 10, Plain shows the significant peaks of CaCO_3_ and Ca(OH)_2_ in the 25°–30° and 15°–20° ranges, respectively. In particular, these indicate that the healing products, which were formed on the crack faces, were mainly CaCO_3_ because the peak of CaCO_3_ was remarkable. Granule-EA shows the peaks of ettringite, CaSO_4_, Ca(OH)_2_, and gypsum. Among them, ettringite and Ca(OH)_2_ may be formed by hydrating the CSA-based expansive agent. CaCO_3_ may be formed by the reaction with CO_2_, as shown in Plain. The peak of gypsum may be supposed to be one of the main ingredients of the CSA-based expansive agent. In particular, the peak of ettringite was remarkable. Therefore, this indicates that the healing products that were formed on the crack faces were mainly ettringite, and that crack closing could be additionally achieved by CaCO_3_ and Ca(OH)_2_.

Finally, Granule-Cm showed the peaks of C–S–H, C–A–H, ettringite, Ca(OH)_2_, etc., which were formed by cement hydration. The peak of CaCO_3_ was also shown in Plain and Granule-EA. This can be supposed to mean that CaCO_3_ was formed by the reaction between Ca(OH)_2_, CO_2_, and sodium carbonate. The peak of gypsum was included in the cement and the CSA-based expansive agent. Therefore, this indicates that the healing products that were formed on the crack faces were mainly hydrates formed by cement hydration, and that ettringite was formed by the CSA-based expansive agent. In addition, it can be supposed that crack closing was slightly achieved via CaCO_3_. In the case of cement, stable hydrate products were created, but they slowed down the generation rate, whereas in the case of the expansive agents, the generation rate of hydrate products such as ettringite was rapidly presented, but it had an unstable condition. Therefore, Granule-Cm#2 indicated a good result because the characteristics of both materials might have reacted well compared to the other mixtures.

Most of the existing relevant literature claims that the primary mechanism of self-healing is the crystallization of calcium carbonate and calcium hydroxide [33]. This view is supported by the fact that precipitated calcium carbonate can often be observed at the outside surfaces of the crack, as a white residue. As one of the cement hydration products dissolved in water, calcium hydroxide is liberated and dissipated along the cracking surfaces, after which the free calcium ions from the cement hydration react with the dissolved CO_2_. Consequently, crystals are formed, growing at both surfaces of the cracks and finally filling the gaps [34].

The CSA-based expansive agent consists of hauyne (3CaO·3Al_2_O_3_·CaSO_4_), free lime (CaO), and anhydrite (CaSO_4_). They mainly provide ettringite (3CaO·Al_2_O_3_, 3CaSO_4_·32H_2_O), a micrometer-sized acicular crystal, due to hydration. In particular, ettringite fills the micropores of concrete and induces its expansion. Also, the CSA-based expansive agent formed crystalline calcium hydroxide via the reaction between free lime (CaO) and water, after which the expansion occurred due to the growth of crystalline calcium hydroxide. Thus, the CSA-based expansive agent caused the expansion induced by the ettringite and calcium hydroxide that were formed due to the hydration reaction. Also, when the cement reacted with the water, various hydration products, such as C–S–H, C–A–H, CH, and ettringite, were formed, and insoluble calcium carbonate (CaCO_3_) was formed by the reaction between sodium carbonate (Na_2_CO_3_) and calcium hydroxide [Ca(OH)_2_], which was in turn formed by C3S and β-C_2_S hydration, and from the CSA-based expansive agent.

Ettringite is a needle-shaped prismatic crystal. It is seen in SEM micrographs in the literature of very young pastes as needles growing into the capillary pores between the cement particles [35]. Ca(OH)_2_ tends to form large crystals with a distinctive hexagonal-prism morphology, but it may vary in morphology as it is found as small equidimensional crystals; large, flat, platy crystals; large, thin, elongated crystals; and all variations in between. The morphology of CaCO_3_ is similar to that of Ca(OH)_2_, and it is mainly shown as having a short hexagonal-prism morphology or a layer morphology. If the hydration further progresses, a membrane-shaped C–S–H will be formed around the particles. This membrane will be dense because it will become thick both inside and outside the particles over time. A C–S–H with a chestnut morphology is formed around the cement particles [36].

**Figure 11:**
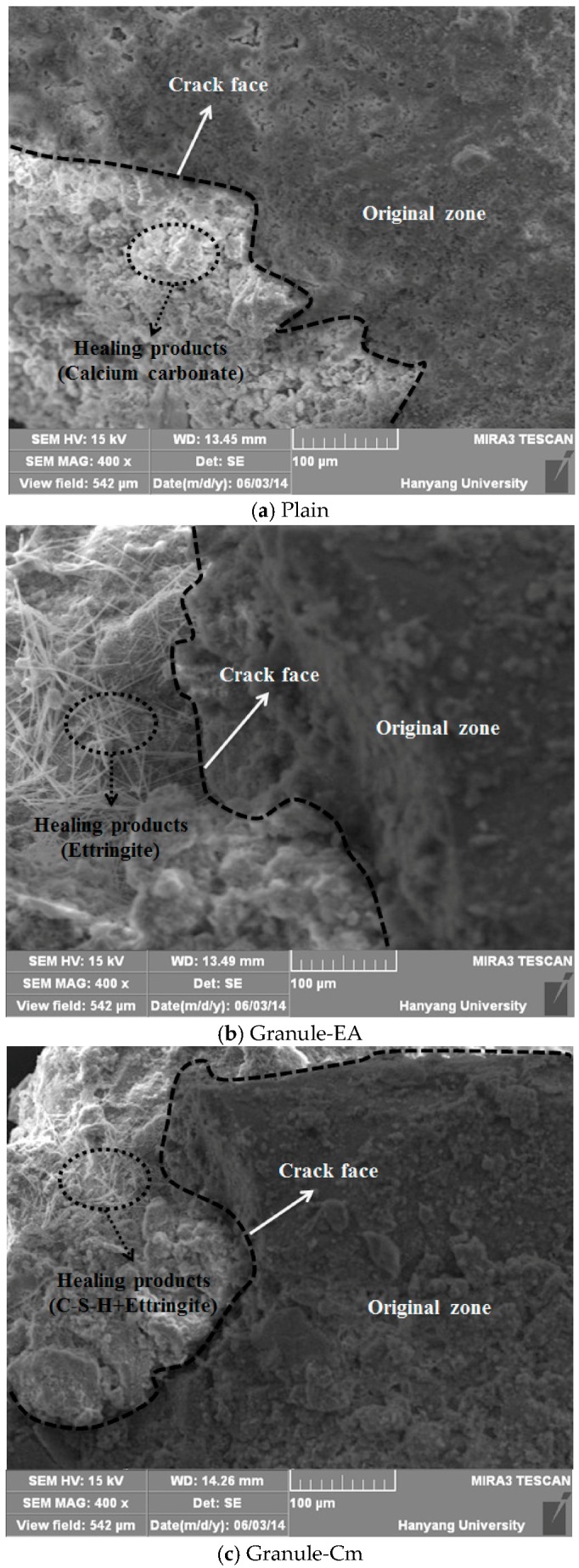
SEM micrographs of the healing products that were formed at the crack face (×400): (**a**) Plain; (**b**) Granule-EA; and (**c**) Granule-Cm.

Figure 11 shows the SEM micrographs of the healing products of Plain, the specimens that incorporated the granulated CSA-based expansive agent with the PVA film coating (Granule-EA), and the granulated cementitious material (the cement + CSA-based expansive agent + sodium carbonate) with the PVA film coating (Granule-Cm). As shown in Figure 11, the crack face was clearly observed, and the original and healing zones can be distinguished from each other. CaCO_3_, which was indicated as having a short hexagonal-prism morphology or a layer morphology, was seen at the healing zone in the SEM micrographs of Plain. Also, in the SEM micrographs of Granule-EA, ettringite, a needle-shaped prismatic crystal, and CaCO_3_, a short hexagonal-prismatic crystal, were seen at the healing zone. Finally, in Granule-Cm, acicular ettringite was visible in the form of a spider web. Also, ettringite had become entangled with the C–S–H gel that was formed as a result of the hydration of the cement particles. Therefore, it was verified that crack closing was achieved by the healing products, as summarized in Table 3.

The CSA-based expansive agent, cement, and sodium carbonate used as healing agents form various hydrates when they react with water. The CSA-based expansive agent causes the expansion induced by ettringite and calcium hydroxide, which are formed due to the hydration reaction. Calcium carbonate (CaCO_3_) is formed by the reaction between sodium carbonate (Na_2_CO_3_) and calcium hydroxide (Ca(OH)_2_). As is well known, cement compounds (C_3_S, C_2_S, C_3_A, and C_4_AF) form hydrates with low solubility with the elapsed time. Among the cement compounds, alite (C_3_S) and belite (C_2_S) form calcium hydroxide and calcium silicate hydrate via hydration reaction. Aluminate (C_3_A) forms calcium aluminate hydrate (C_4_AH_10_ and C_2_AH_8_) through hexagonal crystallization, and forms ettringite by reacting with gypsum. C_4_AF forms ettringite through circular crystallization by reacting with gypsum, which is supplied to sulfate ion. If sulfate ion is not supplied, it is transformed into monosulfate (3CaO·Al_2_O_3_·CaSO_4_·12H_2_O) [25].

## 4. Conclusions

Granulation/coating methods were applied to healing materials (ordinary Portland cement, CSA-based expansive admixture, and sodium carbonate). The healing efficiency of cementitious materials can be maintained via the PVA film thickness until cracks occur, and healing products will be formed because the materials react with the moisture via the crack faces after the moisture dissolves the water-soluble film. In this study, the healing efficiency was evaluated through testing, and the conclusions shown below were reached.

(1) For the granulated cementitious material with a PVA film coating (Granule-Cm), the <0.1 mm crack widths indicated complete crack closing within 14 days; the 0.1–0.2 mm crack widths, within 17 days; and the >0.2 mm crack widths, within 21 days.
(2) In the process of crack closing via self-healing with regard to the typical specimens Plain and those containing granules coated with PVA (Granule-EA and Granule-Cm), which involved 0.1–0.2 mm crack widths within the crack width range, Plain showed crack closing in some parts of the crack even after 28-day immersion. The specimen incorporated with Granule-EA, however, was observed to have had crystals in the crack, except for some parts, after seven-day immersion, with complete crack closing after 14-day immersion. In the same way, in the specimens incorporating Granule-Cm (Granule-Cm#1, Granule-Cm#2, Granule-Cm#3, Granule-Cm#4, and Granule-Cm#5), the extent of crack closing was smaller than that in Granule-EA after seven-day immersion while complete crack closing was observed after 14- to 17-day immersion.
(3) The internal crack closing was evaluated based on the relative dynamic modulus of elasticity. The dynamic modulus of elasticity of Granule-EA rapidly increased early and then stabilized whereas that of Granule-Cm gradually increased and then stabilized. After the completion of the measurement of the dynamic modulus of elasticity, the occurrence of internal crack closing was examined with a microscope. It was seen that Granule-Cm achieved internal crack closing because marks of the cracks could no longer be observed.
(4) A water passing test was conducted to evaluate the prevention of aggressive agents such as water migration due to crack propagation. After the cracking, a similar water permeability coefficient appeared for each crack width range (<0.1 mm, 0.1–0.2 mm, and >0.2 mm). This verified that, unlike the water permeability coefficient of Granule-EA, that of Granule-Cm gradually decreased up to seven days and then rapidly decreased.
(5) The X-ray diffraction (XRD) patterns of the healing products of Granule-Cm showed the peaks of C–S–H, C–A–H, ettringite, Ca(OH)_2_, etc., which were formed by cement hydration. They also showed that acicular ettringite was visible in the form of a spider web in the scanning electron microscopy (SEM) micrographs. Ettringite had become entangled with the C–S–H gel, which had formed as a result of the hydration of the cement particles.

Therefore, it was verified that the granulated healing material with a water-soluble film coating can maintain the healing efficiency of cementitious materials through the water-soluble film until cracks occur, and can prevent water migration via crack closing.

## Figures and Tables

**Table 1:** Comparison of the microcapsule and expansive agents/mineral admixtures as self-healing materials.

Type	Advantage	Disadvantage
Microcapsule	Releases the healing agent only when needed Responds to many damage locations at the same time Possibly effective in multiple-damage events	Difficulty in preparing and casting the capsules Limited amount of the healing agent Concern over the bond between the capsules and the matrix Negative effect on the mechanical properties of the cement matrix if too many capsules are adopted
Expansive agent and mineral admixture	Good healing efficacy Good compatibility of the generated healing products with the cement matrix	Undesirable expansion if not well treated Non-guarantee that the healing products will be generated only as needed

**Table 2:** Granulation materials and their mixture proportions.

S. No.	Specimens	Material Proportions
1	Plain	CSA (0%), OPC (0%)
2	Granule-EA	CSA (10%), OPC (90%)
3	Granule-Cm#1	CSA (10%), OPC (90%), and Na_2_CO_3_ (1%)
4	Granule-Cm#2	CSA (20%), OPC (80%), and Na_2_CO_3_ (1%)
5	Granule-Cm#3	CSA (30%), OPC (70%), and Na_2_CO_3_ (1%)
6	Granule-Cm#4	CSA (40%), OPC (60%), and Na_2_CO_3_ (1%)
7	Granule-Cm#5	CSA (50%), OPC (50%), and Na_2_CO_3_ (1%)

Notes: CSA = CSA-based expensive agents; OPC = ordinary Portland cement; and Na_2_CO_3_ = sodium carbonate.

**Table 3:** Healing products formed at the crack face.

Type	Healing Products
Plain	Calcium carbonate (CaCO_3_), calcium hydroxide [Ca(OH)_2_]
Granule-EA	Ettringite, calcium carbonate (CaCO_3_), calcium hydroxide [Ca(OH)_2_]
Granule-Cm	C–S–H, C–A–H, ettingite, calcium carbonate (CaCO_3_), calcium hydroxide [Ca(OH)_2_]
